# NHERF2 as a Novel Biomarker for Distinguishing MAC Pulmonary Disease from Tuberculosis Based on Proteome Analysis of Serum Extracellular Vesicles

**DOI:** 10.3390/ijms26031155

**Published:** 2025-01-29

**Authors:** Maiko Naito, Yoshito Takeda, Ryuya Edahiro, Yuya Shirai, Takatoshi Enomoto, Mana Nakayama, Satoshi Nojima, Mari Nogami-Ito, Masahide Mori, Yukihiro Yano, Takanori Matsuki, Hanako Yoshimura, Reina Hara, Makoto Yamamoto, Kentaro Masuhiro, Yujiro Naito, Shohei Koyama, Kota Iwahori, Izumi Nagatomo, Takayuki Shiroyama, Kotaro Miyake, Haruhiko Hirata, Hiroaki Hase, Kazutake Tsujikawa, Koji Ueda, Atsushi Kumanogoh

**Affiliations:** 1 Department of Respiratory Medicine and Clinical Immunology, Graduate School of Medicine, Osaka University, Osaka 565-0871, Japan; 2 Department of Immunopathology, World Premier International Research Center Initiative (WPI), Immunology Frontier Research Center (IFReC), Osaka University, Osaka 565-0871, Japan; 3 Center for Advanced Modalities and DDS (CAMaD), Osaka University, Osaka 565-0871, Japan; 4 Department of Statistical Genetics, Graduate School of Medicine, Osaka University, Osaka 565-0871, Japan; 5 Laboratory for Systems Genetics, RIKEN Center for Integrative Medical Sciences, Kanagawa 230-0045, Japan; 6 Department of Pathology, Graduate School of Medicine, Osaka University, Osaka 565-0871, Japan; 7 Compound Library Screening Center, Graduate School of Pharmacological Sciences, Osaka University, Osaka 565-0871, Japan; 8 Department of Respiratory Medicine, NHO Osaka Toneyama Medical Center, Osaka 560-8552, Japan; 9 Laboratory of Molecular and Cellular Physiology, Graduate School of Pharmaceutical Sciences, Osaka University, Osaka 565-0871, Japan; 10 Cancer Proteomics Group, Cancer Precision Medicine Center, Japanese Foundation for Cancer Research, Tokyo 135-8550, Japan; 11 Center for Infectious Diseases for Education and Research (CiDER), Osaka University, Osaka 565-0871, Japan; 12 Integrated Frontier Research for Medical Science Division, Institute for Open and Transdisciplinary Research Initiatives (OTRI), Osaka University, Osaka 565-0871, Japan; 13 Japan Agency for Medical Research and Development—Core Research for Evolutional Science and Technology (AMED–CREST), Osaka University, Osaka 565-0871, Japan

**Keywords:** nontuberculous mycobacteria, tuberculosis, extracellular vesicle, NHERF2

## Abstract

Nontuberculous mycobacterial pulmonary disease (NTM-PD), mainly caused by *Mycobacterium avium* complex (MAC), and pulmonary tuberculosis (TB) are emerging health problems worldwide. However, because their clinical features are often similar, it remains difficult to differentiate NTM-PD from TB when the diagnosis cannot be made by sputum culture. To investigate potential serum biomarkers, we conducted non-targeted proteome analysis on serum extracellular vesicles (EVs) collected from 10 patients with MAC pulmonary disease (MAC-PD), 7 patients with TB, and 10 healthy controls. A total of 2614 proteins were identified in the discovery cohort. The EV protein signature from patients with NTM-PD and TB reflected infectious diseases and inflammatory response pathways. Among the identified proteins, the expression of Na^+^/H^+^ exchanger regulatory factor 2 (NHERF2) was significantly elevated in patients with MAC-PD compared with healthy controls and patients with TB. Moreover, upregulation of NHERF2 was confirmed by immunoblotting of serum EVs and immunohistochemistry of lungs with mycobacterial infection. Our findings highlight that NHERF2 in serum EVs might be a potential biomarker for distinguishing MAC-PD from TB, possibly reflecting the pathogenesis of MAC-PD.

## 1. Introduction

Pulmonary diseases due to mycobacterial infection cause significant morbidity and mortality in humans. Pulmonary tuberculosis (TB), caused by *Mycobacterium tuberculosis* (*Mtb*), remains the leading cause of death from a single infectious pathogen, with 10.6 million new cases and 1.6 million TB-related deaths in 2021 globally [1,2]. On the other hand, the incidence of nontuberculous mycobacterial pulmonary disease (NTM-PD) is increasing and is becoming a major global public health issue [3]. In Japan, the incidence rate for NTM-PD was 14.7 cases per 100,000 person-years in 2014, which is approximately 2.6 times higher than the incidence rate in 2007 (5.7 cases per 100,000 person-years). Surprisingly, this was higher than the incidence rate for TB in the same year (12.9 cases per 100,000 person-years) [4]. Moreover, in the United States, the annual incidence of NTM-PD was 20.1 cases per 100,000 population (from 2010 to 2019), showing a continuous increase throughout the measured period [5]. Although the incidence rate is lower than that in Asia and North America, the incidence of NTM-PD in Denmark increased between 2000 and 2017 (0.4 to 1.3 cases per 100,000 population) as well [6].

NTM are ubiquitously present in the environment worldwide, including in water, soil, and dust [7]. While over 180 species have been discovered, *Mycobacterium avium* complex (MAC) is the most common cause of NTM-PD, causing chronic and progressive pulmonary disease even in immuno-competent patients [8].

In clinical settings, differentiating NTM-PD from TB is challenging because the clinical symptoms and radiographic findings are often similar among patients with pulmonary mycobacterial disease. In general, definitive diagnosis is based on sputum mycobacterial culture and species identification. However, these methods are time-consuming and require strict laboratory standards. Moreover, it is often difficult to obtain sputum samples from asymptomatic patients and the contamination of environmental NTM species requires careful attention [3,9]. To overcome these difficulties, a novel biomarker to distinguish NTM-PD from TB is needed.

Recent advances in mass spectrometry (MS) have led to a proposed alternative method for verifying candidate biomarker proteins. Because the dynamic range of serum proteins is extremely large [10], it is difficult to detect slight amounts of a protein in serum that might reflect the pathophysiology of a disease. Thus, we focused on extracellular vesicles (EVs) in serum. EVs are small lipid bilayer-enclosed particles containing functional biomolecules such as proteins, lipids, RNAs, and DNAs [11]. They are released from various cell types, reflecting the biological status of their parental cells and play important roles in intracellular communications in physiological and pathological settings. In particular, pleiotropic functions of EVs such as immune response, antigen presentation, and tumor progression have been reported [12,13,14,15]. However, the composition, function, and potential to be a diagnostic biomarker for differentiating MAC pulmonary disease (MAC-PD) from TB of EVs is unclear.

In this study, we performed proteome analysis of serum EVs to explore novel biomarkers that can distinguish MAC-PD from TB.

## 2. Results

### 2.1. Discovery of a Novel Biomarker with Proteome Analysis

**Figure 1:**
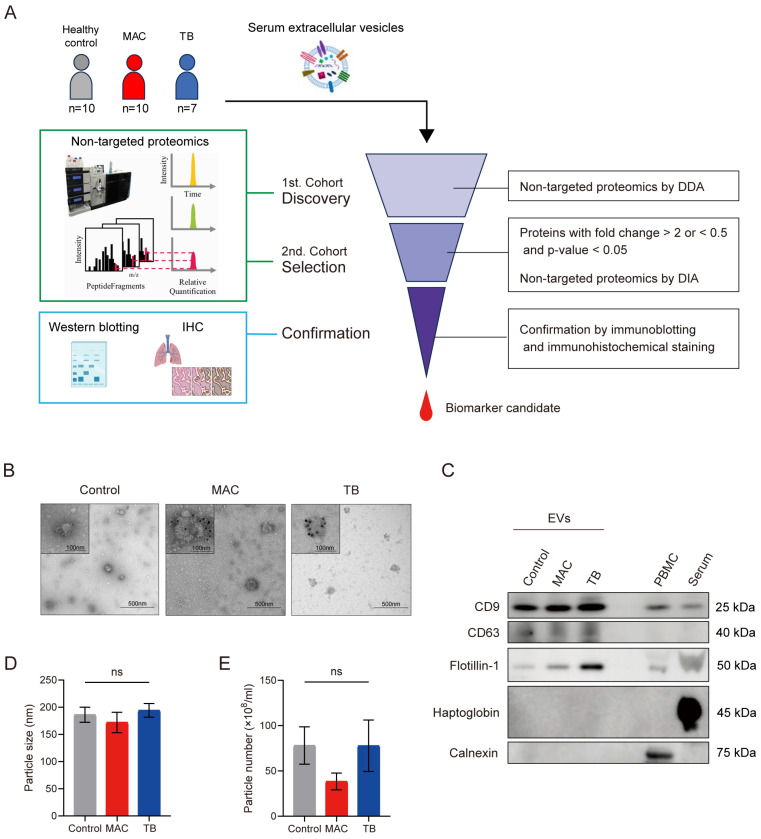
Strategy for the discovery of novel biomarkers to distinguish *Mycobacterium avium* complex pulmonary disease (MAC-PD) from pulmonary tuberculosis (TB). (**A**) In the discovery phase, serum EVs isolated with size-exclusion chromatography from patients with MAC-PD, TB, and healthy controls were analyzed. In the selection phase, biomarker candidates in EVs isolated with ultracentrifugation were analyzed. Finally, biomarkers were confirmed with immunoblotting and immunohistochemical staining. (**B**) Transmission electron microscopy images with immunogold labeling with CD9 of serum EVs from a healthy control, a patient with MAC-PD, and a patient with TB. (**C**) Immunoblotting comparing CD9, CD63, flotillin, haptoglobin, and calnexin levels. (**D**,**E**) No differences were seen in the mean diameter or number of serum EVs from healthy controls versus patients with MAC-PD or TB on NanoSight. Error bars indicate mean ± SD. ns: not significant.

EVs were isolated with size-exclusion chromatography (SEC) from serum samples of control subjects, patients with MAC-PD, and patients with TB (Figure 1A, Table 1). Transmission electron microscopy showed that most of the vesicles expressed CD9, a general EV marker protein, on their surfaces (Figure 1B). In addition, isolated EVs were positive for CD9, CD63, and flotillin-1 and negative for calnexin and haptoglobin (Figure 1C). On the basis of nanoparticle tracking analysis, the size and number of serum EVs from each group were comparable (Figure 1D–E).

**Figure 2:**
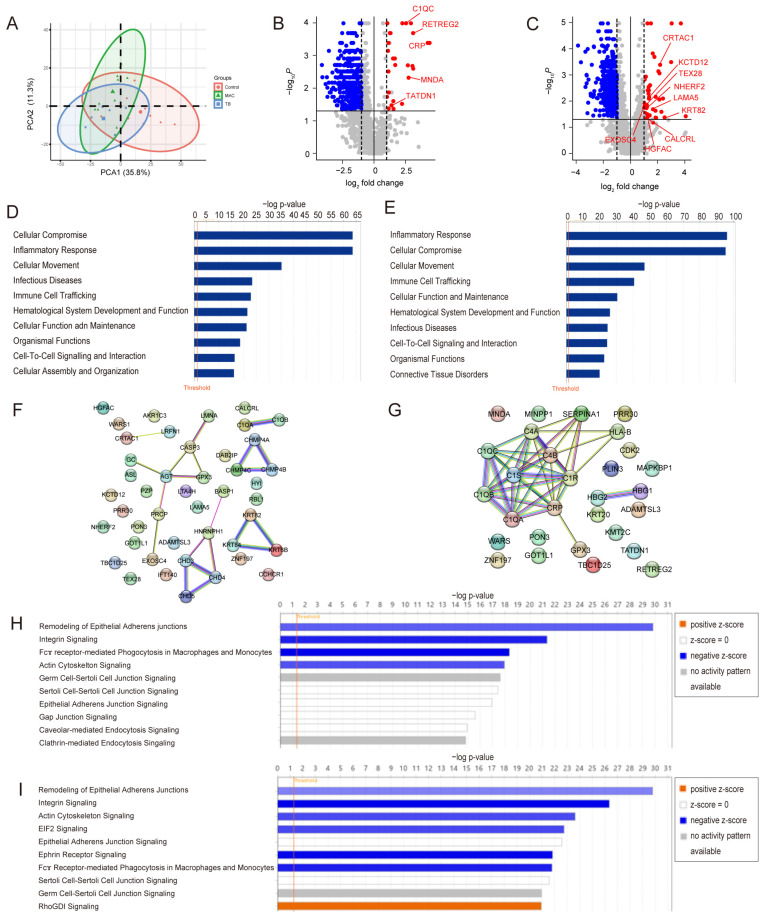
Proteomic profile of serum extracellular vesicles (EVs) reflects mycobacterial infection and its pathogenesis. (**A**) Principal component analysis revealed that proteomic profiling can efficiently discriminate among samples from healthy controls, patients with MAC-PD, and patients with TB. (**B**) A volcano plot of all 2614 serum EV proteins identified in patients with MAC-PD and healthy controls; 44 proteins were significantly upregulated, and 416 proteins were significantly down-regulated in EVs of patients with MAC-PD compared with those of healthy controls. (**C**) A volcano plot of all 2614 serum EV proteins identified in patients with TB and healthy controls; 29 proteins were significantly upregulated, and 548 proteins were significantly down-regulated in EVs of patients with TB compared with those of healthy controls. (**D**) Top 10 disease and function pathways determined by ingenuity pathway analysis to be overrepresented (>2) or underrepresented (<0.5) in non-targeted proteome analyses of serum EVs of patients with MAC-PD compared with those of healthy controls. (**E**) Top 10 disease and function pathways in EVs of patients with TB compared with those of healthy controls. (**F**) Protein–protein interaction network of upregulated proteins in MAC-PD (fold change > 2, *p* < 0.05) constructed with STRING version 12.0. (**G**) Protein–protein interaction network of upregulated proteins in TB (fold change > 2, *p* < 0.05). (**H**) Top 10 canonical pathways identified by IPA for the EV proteins of patients with MAC-PD compared with those of healthy controls. *Blue bars*: negative z-score; *orange bars*: positive z-score; *white bars*: z-score = 0; *gray bars*: no activity pattern available. (**I**) Top 10 canonical pathways identified by IPA for the EV proteins of patients with TB compared with those of healthy controls.

Non-targeted proteome analysis by the data-dependent acquisition (DDA) method identified 2614 proteins in serum EVs. Principal component analysis revealed that proteomic profiling can partially discriminate samples from the different groups (Figure 2A). A comparison of patients with MAC-PD and healthy controls revealed that 44 proteins were significantly upregulated and 416 were down-regulated in patients with MAC-PD (Figure 2B, Table S1). In patients with TB, 29 proteins were significantly upregulated and 548 proteins were down-regulated (Figure 2C, Table S2). Next, we used IPA to explore upstream regulators and perform enrichment analysis (Table S3). From the results from causal analysis in IPA, the altered expression molecules in MAC were mainly regulated by regulatory molecules involved in inflammatory and immune system regulation, such as CD3 (family), IL6, CD40LG, and CCL2. In the case of TB, regulatory molecules involved in inflammatory and immune system regulation, such as PI3K (family), TNF (family), CD3 (family), and CD28, were extracted (Table S4). When compared with control subjects, IPA revealed enrichment of “inflammatory response”, “cellular compromise”, “cellular movement”, “immune cell trafficking”, and “infectious disease” in an analysis of disease and function, among patients with either MAC-PD or TB (Figure 2D,E), which reflected various aspects of inflammation associated with mycobacterial infection. Protein–protein interaction analyses revealed that clusters related to complement components were included among upregulated proteins in both MAC-PD and TB (Figure 2F,G). Down-regulated pathways included “remodeling of the epithelial pathway” and “integrin signaling” in an analysis of the canonical pathway in both MAC-PD and TB when compared with healthy controls (Figure 2H,I).

### 2.2. Selection of a Novel Biomarker Candidate

Upregulated proteins in the disease groups were identified as biomarker candidates. Ten proteins were commonly upregulated in both MAC-PD and TB, nine proteins were upregulated only in MAC-PD, and five proteins were upregulated only in TB (Table 2).

**Figure 3:**
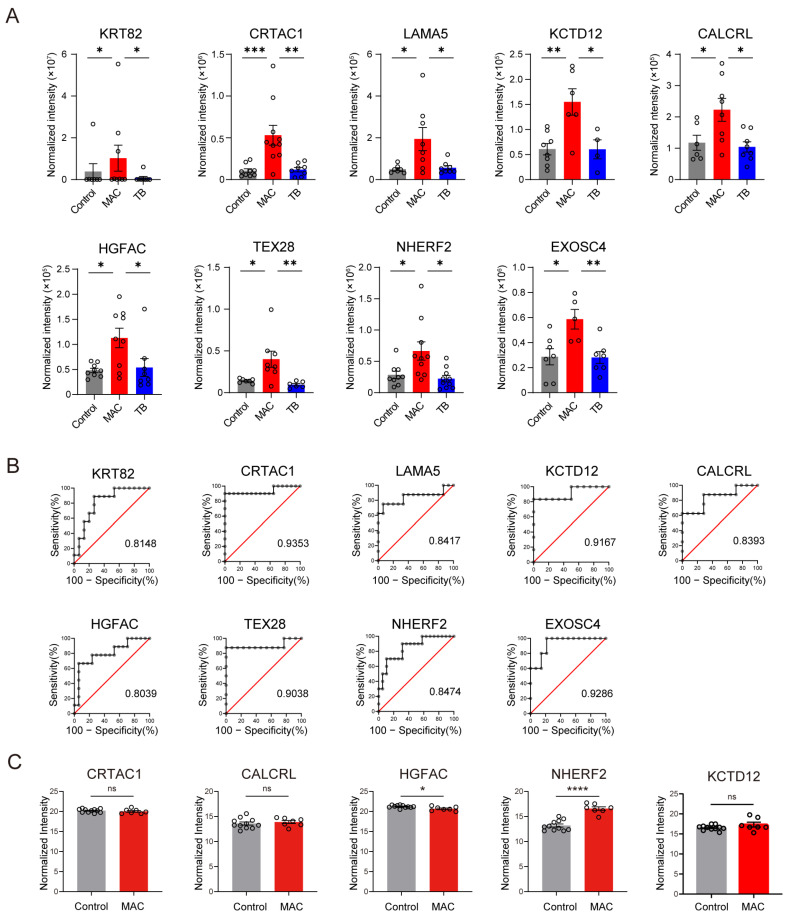
Selection of biomarker candidate proteins. (**A**) The expression of nine specifically upregulated proteins in serum EVs of patients with MAC-PD compared with healthy controls and patients with TB in the first cohort, isolated with size-exclusion chromatography. (**B**) ROC analysis of the nine proteins in A. (**C**) The expression of five proteins identified in serum EVs of patients with MAC-PD and controls in the second cohort, isolated with ultracentrifugation. *Red bars*: MAC-PD; *blue bars*: TB; *gray bars*: healthy control. * *p* < 0.05; ** *p* < 0.01; *** *p* < 0.001.

Considering that the incidence of NTM-PD is increasing worldwide, we focused on proteins upregulated only in MAC-PD. Nine proteins including keratin, type II cuticular Hb2 (KRT82), cartilage acidic protein 1 (CRTAC1), laminin subunit alpha-5 (LAMA5), BTB/POZ domain-containing protein KCTD12 (KCTD12), calcitonin gene-related peptide type 1 receptor (CALCRL), hepatocyte growth factor activator (HGFAC), testis-specific protein TEX28 (TEX28), Na^+^/H^+^ exchange regulatory cofactor NHE-RF2 (NHERF2), and exosome complex component RRP41 (EXOSC4) were upregulated in patients with MAC-PD, but not in patients with TB (Figure 3A). ROC analysis revealed that all nine proteins are potential diagnostic serum EV markers for MAC-PD with AUC > 0.80 (Figure 3B).

For biomarker selection, we conducted non-targeted proteome analysis by the data-independent acquisition (DIA) method on serum EVs isolated with ultracentrifugal separation in the second cohort consisting of 11 healthy controls and 7 patients with MAC-PD (Table 3). Among the nine proteins identified as biomarker candidates for MAC-PD in the discovery cohort, CRTAC1, CALCRL, HGFAC, NHERF2, and KCTD12 were detected in the selection cohort. When compared with healthy controls, NHERF2 was the only protein that was upregulated in patients with MAC-PD (Figure 3C).

### 2.3. Confirmation of a Novel Biomarker

**Figure 4:**
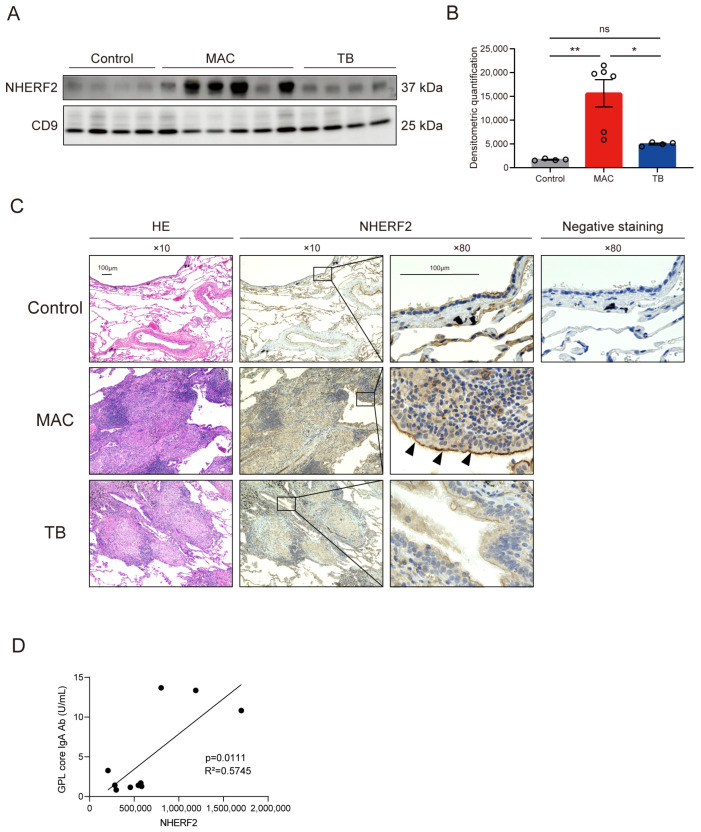
Confirmation of NHERF2 upregulation in serum EVs and lungs of patients with MAC-PD or TB. (**A**) A representative immunoblotting comparing NHERF2 in serum EVs isolated with size-exclusion chromatography of healthy controls and patients with MAC-PD or TB. (**B**) Densitometric analysis of the immunoblotting results in B. (**C**) A representative image of immunohistochemistry results for NHERF2 in lung sections from controls, patients with MAC-PD, and patients with TB. NHERF2 was highly expressed in ciliated cells (black arrow heads) of a patient with MAC-PD. (**D**) Linear regression model comparing GPL core IgA antibody levels (x-axis) and NHERF2 levels (y-axis). The coefficients of determination (r^2^) are indicated. * *p* < 0.05; ** *p* < 0.01.

The presence and upregulation of NHERF2 were subsequently confirmed by Western blotting. Densitometric relative quantification of the immunoblotting results revealed a significant increase in NHERF2 levels in serum EVs of patients with MAC-PD but not in those of patients with TB (Figure 4A,B).

To further confirm these results, we performed immunohistochemistry of resected human lung pathology specimens. In normal lung tissue, NHERF2 was expressed in the apical plasma membrane of epithelial cells and endothelial cells, including cells in the epithelium of bronchioles, ciliated cells, pneumocytes, and cells in the vascular endothelium, as reported previously [16]. Lung specimens of a patient with MAC-PD had enhanced expression of NHERF2 in these cells when compared with controls and patients with TB (Figure 4C).

To characterize the clinical potential of NHERF2, we analyzed the association between NHERF2 and serum anti-glycopeptidolipid (GPL) core IgA antibody, which is supplementarily measured for the clinical diagnosis of MAC-PD [17]. Notably, NHERF2 levels were significantly correlated with GPL core IgA antibody levels (*p* = 0.0111, Figure 4D).

## 3. Discussion

The present study using serum EV proteome analysis identified NHERF2 as a potential biomarker for distinguishing MAC-PD from TB. In addition, the enhanced expression of NHERF2 was pathologically confirmed in resected lung tissue from patients with MAC-PD but not TB, suggesting its pathophysiological role in MAC-PD.

In the context of mycobacterial infectious disease, both host- and pathogen-derived-EVs have emerged as a focus of study. Detection of pathogen-derived EVs in a host specimen is helpful for the diagnosis of infection by the pathogen detected. *Mtb* peptides have been found in serum EVs of patients with TB [18] and, moreover, in latent TB [19]. In addition, *Mycobaceterium avium* (*M. avium*) GPLs are released from infected macrophages [20], indicating their potential as diagnostic markers. The comprehension of infected host-derived EVs contributes to the understanding of mycobacterial infection and its pathology; it is also useful for biomarker-based diagnostics. EVs from *Mtb*-infected macrophages activate endothelial cells [21]. EVs from macrophages infected with M. *avium* stimulate a proinflammatory response in mouse bone marrow-derived macrophages [20]. Consistent with our results, comparative proteome analysis of plasma EVs revealed that the complement pathway and coagulation are enriched in patients with mycobacterial infection, including *Mycobacterium abscessus*, MAC, and *Mtb* [22]. Although studies reporting the composition and function of host-derived EVs in mycobacterial infection are accumulating, there have been no comparative analyses of serum EV proteins from patients with MAC-PD and TB to explore biomarkers for distinguishing between these two diseases. This is the first comprehensive comparative proteome analysis of EVs from patients with MAC-PD or TB, showing the potential ability of NHERF2 to act as a marker for the pathophysiological state of MAC-PD.

A serodiagnosis enzyme immunoassay kit for detecting anti-GPL core IgA antibody is commercially available. It has good sensitivity and specificity for the diagnosis of MAC-PD [17]. GPL core antibody levels are associated with disease activity and treatment outcomes in patients with MAC-PD [23]. We found that NHERF2 levels in serum EVs are associated with GPL core antibody levels in patients with MAC-PD, suggesting that NHERF2 might be useful for diagnosis, disease monitoring, and prognostication. However, interpretation of GPL core antibody results requires careful consideration in immuno-compromised hosts, who have suppressed antibody production [24]. Because NHERF2 production is unaffected by the ability to produce antibodies, NHERF2 might be effective for the diagnosis of MAC-PD in immuno-compromised patients.

NHERF2 localizes to the apical plasma membrane of epithelial cells and acts as a protein adapter. The NHERF2 protein contains two tandem postsynaptic density-95, discs large, zona occludens-1 (PDZ) domains and a C-terminal sequence that binds several members of the ezrin–radixin–moesin (ERM) family of membrane cytoskeletal adapters [25]. These structures are needed to bind membrane and non-membrane proteins together, leading to its role as a regulator of the Na^+^/H^+^ exchanger (NHE) and cystic fibrosis transmembrane conductance regulator (CFTR) [26].

NTM-PD commonly occurs in patients with preexisting lung diseases such as chronic obstructive pulmonary disease, bronchiectasis, cystic fibrosis (CF), and pneumoconiosis, in addition to post-menopausal women without clearly recognized lung disease [3]. Mucociliary clearance is an important factor in the development of NTM-PD, because CF and genetic variants of its causative gene CFTR are associated with NTM-PD [27]. CFTR is a chloride channel expressed in epithelial cells that regulates chloride and water transport. Dysfunction of CFTR makes airway mucus viscous, leading to decreased mucociliary clearance and repeated chronic airway infections [28]. The function of CFTR is regulated by dynamic protein–protein interactions including CFTR–NHERF2–lysophosphatidic acids receptor 2 (LPA_2_) complex [29]. NHERF2 is required for the LPA_2_-mediated inhibition of CFTR [30]. In addition, in patients with class II CF mutations, *NHERF2* gene expression is upregulated [31]. These previous studies suggest that NHERF2 plays a crucial role in suppressing CFTR function, and its upregulation might be related to CFTR dysfunction. However, to prove the role of the upregulation of NHERF2 in controlling CFTR function, more mechanistic studies are needed.

NHERF2 was initially described as an NHE regulator. NHERF2 is an essential component in the NHE3/ezrin/cAMP dependent protein kinase 2 multiprotein signaling complex, which is required for the inhibition of ion transport via the phosphorylation of NHE3 [32]. Recently, a genome-wide association study of patients with MAC-PD identified disease risk associated with SNPs in the *Calcineurin B homologous protein 2* (*CHP2)* region, which is a cofactor for NHEs [33]. These findings suggest the importance of NHEs in NTM infection.

Existing failure of mucociliary clearance can induce NTM infection, but NTM infection itself can damage cilia and affect mucociliary clearance in airways [34]. Dysfunction of ion transporters, including CFTR and NHE, might contribute to the suppression of airway clearance due to NTM infection. Moreover, although not clearly proven, elevated NHERF2 protein levels in serum EVs from patients with MAC-PD might reflect the effects of pathways suppressing mucociliary clearance. Further study is warranted to investigate the interaction between NTM infection and airway epithelial ion transporters.

This study has several limitations. First, the sample size of this study was relatively small to prove the reliability and specificity of NHERF2 as a diagnostic biomarker for MAC-PD. To consider the usefulness of this protein as a clinical biomarker, it is necessary to confirm the results in a larger and more diverse cohort. Second, because MAC is the most common causative species of NTM, our study focused on MAC and excluded other NTM pathogens. NTM-PD caused by species other than MAC might have different clinical characteristics and might not present increased expression of NHERF2.

In conclusion, we identified NHERF2 as a potential biomarker for distinguishing MAC-PD from TB. NHERF2 might be associated with the pathogenesis of MAC-PD via regulation of ion transporters. Large-scale studies are needed to further validate the diagnostic significance of NHERF2 in serum EVs. Further studies will clarify the function of NHERF2 in MAC-PD.

## 4. Materials and Methods

### 4.1. Study Design

The 1st cohort, as a discovery cohort, consisted of 10 control subjects, 10 patients with MAC-PD, and 7 patients with TB (Table 1). MAC-PD and TB were diagnosed according to the American Thoracic Society and Infectious Diseases Society of America criteria, respectively [3,9]. Patients with MAC-PD and TB were untreated at the time of sample collection. EV samples isolated with size-exclusion chromatography (SEC) were subjected to quantitative high-throughput proteome analysis with liquid chromatography–mass spectrometry (LC-MS/MS) and analyzed by the data-dependent acquisition (DDA) method (Table S5). The 2nd cohort for the selection of candidate proteins included 11 control subjects and 7 patients with MAC-PD (Table 3). Candidate proteins identified in the discovery cohort were quantified using LC-MS/MS of EV samples isolated with ultracentrifugation and analyzed by the data-independent acquisition (DIA) method (Table S6).

### 4.2. Sample Collection and EV Isolation

All serum samples were collected in Osaka University Hospital or Toneyama Medical Center and stored at −80 °C until analysis. During the discovery phase, serum EVs were isolated with SEC, using an EV Second L70 SEC column (GL Science, Tokyo, Japan), according to the manufacturer’s instructions. During the selection phase, serum EVs were isolated with ultracentrifugation using differential cushions as described previously [15]. The isolation of EVs was confirmed using the guidelines delineated in the Minimal Information for Studies of Extracellular Vesicles 2023 guidelines (MISEV2023) [13]. Size distributions and numbers were confirmed by NanoSight nanoparticle tracking analysis (Malvern Instruments, Worcestershire, UK).

### 4.3. Transmission Electron Microscopy

EV samples were absorbed onto a formvar/carbon-coated nickel grid for 1 h. EVs were fixed with 2% paraformaldehyde and then incubated with the following primary antibodies: anti-CD9 (MM2/57; Thermo Fisher Scientific, Waltham, MA, USA). Immunoreactive EVs were visualized using anti-mouse IgG(H+L) (EMGMHL10) and anti-rabbit IgG(H+L) (EMGFAR10; BBI Solutions, Gwent, UK) antibodies preabsorbed with 10 nm gold particles.

### 4.4. Protocol for Data-Dependent Acquisition

EVs were dissolved in 30 μL of 1 × Laemmli’s sample buffer. After reduction with 10 mM TCEP at 100 °C for 10 min and alkylation with 50 mM iodoacetamide at ambient temperature for 45 min, protein samples were subjected to SDS-PAGE. The electrophoresis was stopped at the migration distance of 2 mm from the top edge of the separation gel. After CBB-staining, protein bands were excised, destained, and cut finely prior to in-gel digestion with Trypsin/Lys-C Mix (Promega, Madison, WI, USA) at 37 °C for 12 h.

The resulting peptides were extracted from gel fragments and analyzed with an Orbitrap Fusion Lumos mass spectrometer (Thermo Scientific, Waltham, MA, USA) combined with an UltiMate 3000 RSLC nano-flow HPLC (Thermo Scientific, Waltham, MA, USA). Peptides were enriched with μ-Precolumn (0.3 mm i.d. × 5 mm, 5 μm, Thermo Scientific, Waltham, MA, USA) and separated on an AURORA column (0.075 mm i.d. × 250 mm, 1.6 μm, Ion Opticks Pty Ltd., Victoria, Australia) using the two-step gradient: 2–40% acetonitrile for 110 min, followed by 40–95% acetonitrile for 5 min in the presence of 0.1% formic acid. The analytical parameters of Orbitrap Fusion Lumos were set as follows: resolution of full scans = 50,000, scan range (*m*/*z*) = 350–1500, maximum injection time of full scans = 50 msec, AGC target of full scans = 4 × 10^5^, dynamic exclusion duration = 30 s, cycle time of data-dependent MS/MS acquisition = 2 s, activation type = HCD, detector of MS/MS = ion trap, maximum injection time of MS/MS = 35 msec, AGC target of MS/MS = 1 × 10^4^.

The MS/MS spectra were searched against the Homo sapiens protein sequence database (Nov. 2017, 20,366 entries) in Uniprot using Proteome Discoverer 2.4 software (Thermo Scientific, Waltham, MA, USA), in which peptide identification filters were set at “false discovery rate < 1%”. Label-free relative quantification analysis for proteins was performed with the default parameters of Minora Feature Detector node, Feature Mapper node, and Precursor Ions Quantifier node in Proteome Discoverer 2.4 software.

### 4.5. Protocol for Data-Independent Acquisition

To prepare the EV suspension for analysis, sodium deoxycholate was added to a final concentration of 1%, and lysis was performed. The lysate was treated with 10 mM tris(2-carboxyethyl) phosphine (TCEP) at 55 °C for 1 h to reduce disulfide bonds. Following this, iodoacetamide was added at a final concentration of 17 mM for alkylation, which was carried out in the dark at room temperature for 30 min. The samples underwent purification via methanol–chloroform treatment and were then reconstituted in 50 mM ammonium bicarbonate solution. Proteins were digested overnight at 37 °C with trypsin at a concentration of 0.05 µg/µL. Online liquid chromatography–mass spectrometry (LC-MS) was performed using an Easy-nLC 1200 system coupled to an Orbitrap Eclipse Tribrid mass spectrometer (Thermo Fisher Scientific, Waltham, MA, USA). The peptides were first trapped on a C18 guard-desalting column (Acclaim PepMap 100, 75 µm × 2 cm, nanoViper, C18, 5 µm, 100 Å) and then separated on a 12 cm analytical C18 column (C18, 3 µm, 75 µm × 12 cm; Nikkyo Technos, Tokyo, Japan). The nano capillary gradient solvent A consisted of 0.1% formic acid in water, while solvent B consisted of 80% acetonitrile, 20% water, and 0.1% formic acid. The gradient was executed at a constant flow rate of 0.3 µL/min, starting from 6% solvent B, increasing to 31% over time, and ending with a sharp rise to 90% within 10 min. For data-independent acquisition (DIA), MS1 spectra were acquired with an m/z range of 500–1100 at a resolution of 24,000. The automatic gain control (AGC) target was set to 500%, with a maximum injection time of 50 milliseconds, and the data were recorded in centroid mode. MS2 spectra were obtained in quadrupole isolation mode using a window size of 10 m/z. High-energy collision dissociation (HCD) was applied with a collision energy of 25%. The resolution for MS2 spectra was set to 120,000, the AGC target was adjusted to 2000%, and the data were also recorded in centroid mode. The raw data were analyzed using DIA-NN software (version 1.8.1) in a library-free mode. For peptide identification, trypsin specificity was maintained, allowing one missed cleavage. Variable modifications included N-terminal methionine removal, and fixed modifications were set as carbamidomethylation on cysteine residues. The precursor mass range was set between 300 and 1800 *m*/*z*, with fragment ions ranging from 200 to 1800 *m*/*z*. Both MS1 and MS2 mass accuracies were automatically determined. The “Protein names (from FASTA, Aug 2022)” option was selected for protein inference, alongside the Heuristic inference and Match Between Runs (MBR) features. The quantification process utilized retention time (RT)-dependent and high-precision robust LC settings.

### 4.6. Bioinformatic Analysis of the Proteome

To elucidate biologically relevant proteomic pathways and molecular networks, we used ingenuity pathway analysis (IPA; Qiagen, Venlo, Netherlands.) for upstream and enrichment analyses.

### 4.7. Western Blotting

EVs isolated from 350 μL of serum with SEC were lysed in a radioimmunoprecipitation assay buffer (Ref 89900; Thermo Fisher Scientific, Waltham, MA, USA) with a protease inhibitor cocktail (Cat A-0014; ITSI Biosciences, Johnstown, PA, USA). Protein samples were loaded onto NuPAGE 4–12% Bis-Tris gels (Invitrogen, Waltham, MA, USA). For immunoblotting analysis, gels were electroblotted onto polyvinylidene difluoride membranes (Bio-Rad, Hercules, CA, USA). Membranes were blocked with Blocking One (Nacalai Tesque, Kyoto, Japan), incubated with a specific primary antibody, and then incubated with the appropriate secondary antibody.

The following primary antibodies were used for immunoblotting: mouse anti-human NHERF2 (ab151443; Abcam, Cambridge, UK), mouse anti-human flotillin-1 (610821; BD Biosciences, Franklin Lakes, NJ, USA), mouse anti-human CD9 (MM2/57; Thermo Fisher Scientific, Waltham, MA, USA), mouse anti-human CD63 (MEX002-3; Medical & Biological Laboratories, Tokyo, Japan), rabbit anti-human calnexin (ab22595; Abcam, Cambridge, UK), and rabbit anti-human haptoglobin (ab131236; Abcam, Cambridge, UK). Immunoreactive signals were visualized using the SuperSignal West Femto Maximum Sensitivity Substrate (Thermo Fisher Scientific, Waltham, MA, USA) and detected on an ImageQuant LAS500 (GE Healthcare, Chicago, IL, USA). ImageJ (National Institutes of Health) was used for densitometric analysis.

### 4.8. Immunohistochemistry

Paraffin-fixed lung tissue samples obtained from surgical specimens at Osaka University Hospital were used for immunostaining. Immunohistochemical staining of these samples were performed by Applied Medical Research Laboratory (Osaka, Japan). After deparaffinization of the paraffin-fixed tissues, antigen retrieval was performed by autoclaving for 15 min at 125 °C in an EDTA buffer solution (pH 9). Endogenous peroxidase activity was blocked with 3% bovine serum albumin in phosphate-buffered saline at room temperature for 1 h. Slides were incubated with NHERF2 Polyclonal Antibody (PA5-84728; Invitrogen, Waltham, MA, USA) at 4 °C overnight, followed by incubation with horseradish peroxidase-conjugated anti-rabbit secondary antibody (02-6102; Invitrogen, Waltham, MA, USA) at room temperature for 30 min.

### 4.9. Statistical Analysis

All statistical analyses were performed using GraphPad Prism version 10 (GraphPad Software) or R (version 4.3.1). The relative abundance of proteins identified in the non-targeted proteome analysis was compared using the Wilcoxon signed-rank test. Statistical comparisons between two groups were performed using the non-paired, two-tailed *t*-test. A one-way ANOVA with the Tukey–Kramer post hoc test was used to compare more than two groups. Results are shown as means ± SEM. Fold change cutoff was >2 or <0.5. Results were considered statistically significant at *p* < 0.05.

## Figures and Tables

**Table 1:** Patient characteristics at enrolment (first cohort).

	Healthy Control	MAC	TB
n	10	10	7
Age, y	47.8 ± 9.53	62.8 ± 6.69	42 ± 10.6
Male/Female	6(60)/4(40)	0(0)/10(100)	5(71)/2(29)
BMI	23.0 ± 2.14	20.0 ± 1.48	20.3 ± 1.42

BMI; body mass index, MAC; *Mycobacterium avium* complex, TB; tuberculosis. Data represent number (%) or mean (+/− standard deviation).

**Table 2:** Significantly upregulated proteins in serum extracellular vesicles of patients with MAC and TB. MAC; *Mycobacterium avium* complex, TB; tuberculosis, HC; healthy control.

Upregulated only in MAC								
Protein ID	Gene	Protein name	Fold change (MAC vs. HC)	*p*-value (MAC vs. HC)	Fold change (TB vs. HC)	*p*-value (TB vs. HC)	Fold change (MAC vs. TB)	*p*-value (MAC vs. TB)
Q9NSB4	KRT82	Keratin, type II cuticular Hb2	5.72	0.04178	1.48	0.62821	3.86	0.01758
Q9NQ79	CRTAC1	Cartilage acidic protein 1	4.55	0.00041	1.32	0.95465	3.46	0.00300
O15230	LAMA5	Laminin subunit alpha-5	3.16	0.02051	0.85	0.80478	3.74	0.02051
Q96CX2	KCTD12	BTB/POZ domain-containing protein KCTD12	2.56	0.00799	1.07	1.00000	2.39	0.03810
Q16602	CALCRL	Calcitonin gene-related peptide type 1 receptor	2.35	0.04262	0.84	0.69913	2.80	0.01998
Q04756	HGFAC	Hepatocyte growth factor activator	2.35	0.03147	0.87	0.60639	2.70	0.02897
O15482	TEX28	Testis-specific protein TEX28	2.31	0.01399	0.65	0.05128	3.54	0.00466
Q15599	SLC9A3R2	Na(+)/H(+) exchange regulatory cofactor NHE-RF2	2.30	0.01721	0.90	0.68059	2.55	0.01357
Q9NPD3	EXOSC4	Exosome complex component RRP41	2.01	0.01768	1.01	0.87626	2.00	0.00794
**Upregulated only in TB**								
Protein ID	Gene	Protein name	Fold change (MAC vs. HC)	*p*-value (MAC vs. HC)	Fold change (TB vs. HC)	*p*-value (TB vs. HC)	Fold change (TB vs. MAC)	*p*-value (TB vs. MAC)
P02741	CRP	C-reactive protein	0.73	0.97051	21.57	0.00041	29.57	0.00463
Q8NC44	RETREG2	Reticulophagy regulator 2	0.78	0.66072	8.67	0.00021	11.11	0.00787
P41218	MNDA	Myeloid cell nuclear differentiation antigen	0.99	0.60481	6.76	0.00480	6.86	0.00480
P02747	C1QC	Complement C1q subcomponent subunit C	1.84	0.02323	5.87	0.00010	3.19	0.01851
Q6P1N9	TATDN1	Putative deoxyribonuclease TATDN1	0.99	0.91818	2.63	0.03357	2.65	0.02424
**Upregulated in both MAC and TB**								
Protein ID	Gene	Protein name	Fold change (MAC vs. HC)	*p*-value (MAC vs. HC)	Fold change (TB vs. HC)	*p*-value (TB vs. HC)	Fold change (TB vs. MAC)	*p*-value (TB vs. MAC)
P22352	GPX3	Glutathione peroxidase 3	13.12	0.00001	19.40	0.00041	1.48	0.88677
Q15166	PON3	Serum paraoxonase/lactonase 3	7.97	0.00032	2.91	0.02499	0.36	0.10880
Q53SZ7	PRR30	Proline-rich protein 30	4.37	0.00866	8.73	0.00253	2.00	0.18065
Q3MII6	TBC1D25	TBC1 domain family member 25	3.62	0.00684	2.94	0.04309	0.81	0.53620
Q8NHS2	GOT1L1	Putative aspartate aminotransferase, cytoplasmic 2	3.40	0.00021	2.22	0.00309	0.65	0.07024
P82987	ADAMTSL3	ADAMTS-like protein 3	2.84	0.00001	2.24	0.00041	0.79	0.26985
P23381	WARS	Tryptophan--tRNA ligase, cytoplasmic	2.38	0.00001	4.79	0.00010	2.01	0.00679
O14709	ZNF197	Zinc finger protein 197	2.26	0.01851	6.49	0.00200	2.87	0.02424
P02745	C1QA	Complement C1q subcomponent subunit A	2.07	0.01522	8.38	0.00210	4.05	0.09386
P02746	C1QB	Complement C1q subcomponent subunit B	2.00	0.01150	7.69	0.00010	3.84	0.00967

**Table 3:** Patient characteristics at enrolment (second cohort).

	Healthy Control	MAC
n	11	7
Age, y	61.2.8 ± 9.58	67.3 ± 8.38
Male/Female	4(40)/6(60)	2(29)/5(71)
BMI	23.3 ± 3.71	19.0 ± 2.79

BMI; body mass index, MAC; Mycobacterium avium complex. Data represent number (%) or mean (+/− standard deviation).

## Data Availability

The data presented within this study are available upon reasonable request from the corresponding author.

## Supplementary Materials

The following supporting information can be downloaded at: https://www.mdpi.com/article/10.3390/ijms26031155/s1.

## Abbreviations

The following abbreviations are used in this manuscript:

NTM	Nontuberculous mycobacterium
MAC	Mycobacterium avium complex
TB	Tuberculosis
NHERF2	Na+/H+ exchanger regulatory factor 2
